# Dietary Omega-3 Polyunsaturated Fatty Acids Alter the Fatty Acid Composition of Hepatic and Plasma Bioactive Lipids in C57BL/6 Mice: A Lipidomic Approach

**DOI:** 10.1371/journal.pone.0082399

**Published:** 2013-11-21

**Authors:** Kayode A. Balogun, Carolyn J. Albert, David A. Ford, Robert J. Brown, Sukhinder K. Cheema

**Affiliations:** 1 Department of Biochemistry, Memorial University, St. John’s, NL, Canada; 2 Department of Biochemistry and Molecular Biology, and Center for Cardiovascular Research, Saint Louis University, St. Louis, Missouri, United States of America; Wageningen University, Netherlands

## Abstract

**Background:**

Omega (n)-3 polyunsaturated fatty acids (PUFA) are converted to bioactive lipid components that are important mediators in metabolic and physiological pathways; however, which bioactive compounds are metabolically active, and their mechanisms of action are still not clear. We investigated using lipidomic techniques, the effects of diets high in *n*-3 PUFA on the fatty acid composition of various bioactive lipids in plasma and liver.

**Methodology and Principal Findings:**

Female C57BL/6 mice were fed semi-purified diets (20% w/w fat) containing varying amounts of *n*-3 PUFA before mating, during gestation and lactation, and until weaning. Male offspring were continued on their mothers’ diets for 16 weeks. Hepatic and plasma lipids were extracted in the presence of non-naturally occurring internal standards, and tandem electrospray ionization mass spectrometry methods were used to measure the fatty acyl compositions. There was no significant difference in total concentrations of phospholipids in both groups. However, there was a significantly higher concentration of eicosapentaenoic acid containing phosphatidylcholine (PC), lysophosphatidylcholine (LPC), and cholesteryl esters (CE) (*p* < 0.01) in the high *n*-3 PUFA group compared to the low *n*-3 PUFA group in both liver and plasma. Plasma and liver from the high *n*-3 PUFA group also had a higher concentration of free *n*-3 PUFA (*p* < 0.05). There were no significant differences in plasma concentrations of different fatty acyl species of phosphatidylethanolamine, triglycerides, sphingomyelin and ceramides.

**Conclusions/Significance:**

Our findings reveal for the first time that a diet high in *n*-3 PUFA caused enrichment of *n*-3 PUFA in PC, LPC, CE and free fatty acids in the plasma and liver of C57BL/6 mice. PC, LPC, and unesterified free *n*-3 PUFA are important bioactive lipids, thus altering their fatty acyl composition will have important metabolic and physiological roles.

## Introduction

Essential polyunsaturated fatty acids (PUFA) of the omega-3 (*n*-3) and *n*-6 classes are important in the regulation of metabolic processes. N-3 PUFA such as docosahexaenoic acid (DHA; 22:6) and eicosapentaenoic acid (EPA; 20:5) have attracted a lot of attention in the past years as a result of their potential health benefits [1-3]. N-3 PUFA has been shown to prevent atherosclerosis [4,5], regulate nuclear transcription factors involved in gene expression of inflammatory markers, and stimulate cognitive development [6,7]. Markers of cardiovascular disease such as high triglycerides (TG), endothelial dysfunction, cardiac arrhythmia, and inflammation are also reduced by the administration of *n*-3 PUFA [1,8-11]. 

*In vivo*¸ *n*-3 PUFA can either exist as free products of enzyme hydrolysis or bound to phospholipids (PL) or TG. Reports have shown that dietary *n*-3 PUFA are preferentially incorporated into PL compared to TG [12]. PL are important constituents of the cellular membrane bilayer, with phosphatidylcholine (PC) and phosphatidylethanolamine (PE) being the most abundant [13]. Membrane fluidity can be influenced by the incorporation of dietary long chain *n*-3 PUFA into membrane PL, which enhances the functions of transmembrane proteins and their interactions with extracellular ligands [14-16]. This indirectly affects signalling pathways and other physiological functions of the membrane. PC has been implicated in an array of physiological functions, and their functional properties depend on their fatty acyl chains. 

The liver is principally involved in the metabolism and release of PC into circulation. PC is metabolized by the enzyme phospholipase A_2_ releasing the fatty acid at the *sn*-2 position, and lysophosphatidylcholine (LPC) into the plasma pool for distribution to extrahepatic tissues [17,18]. Upon hydrolysis of the *sn*-2 fatty acid of PC, the released free fatty acids (FFA) are further metabolized to form bioactive compounds with either pro-inflammatory or anti-inflammatory properties [18]. Arachidonic acid (AA; 20:4), a long chain *n*-6 PUFA released from the metabolism of PC, is further metabolized by the cyclooxygenase or lipoxygenase enzyme pathways to produce the inflammatory series-2 prostaglandins or series-4 leukotrienes, respectively [19]. On the contrary, the cyclooxygenase and lipoxygenase metabolic products of EPA, an *n*-3 PUFA, are generally anti-inflammatory [20]. Furthermore, protectins and resolvins, the products of oxygenation of DHA and EPA, have been reported to exhibit anti-inflammatory properties [21-23]. 

LPC, the other product of enzyme hydrolysis of PC, is an important lipid mediator involved in cellular metabolism. Direct hepatic secretion is an important source of the abundant unsaturated LPC found in the plasma [24]. LPC has been controversially linked with the development of atherosclerosis [25-27]. However, there are burgeoning evidences suggesting that the biological properties of LPC depend on the acyl chain of the molecule [28,29]. Studies have reported an increase in plasma concentration of saturated LPC in diseases conditions such as obesity, diabetes and rheumatoid arthritis [30]. In another line of evidence, polyunsaturated LPC significantly reversed saturated LPC induced inflammation [31]. However, the importance of specific LPC fatty acyl chain in metabolic regulation is still not clear.

Most studies to date have explored the health benefits of *n*-3 PUFA esterified to ethyl esters or TG [32-34]. Moreover, the majority of the reported findings that support the pro-inflammatory and the atherogenic actions of LPC were established using saturated or monounsaturated LPC [25,26,35-37]. Cells, tissues, and biological fluids consist of numerous bioactive lipid mediators involved in cellular processes, which are likely altered by dietary *n*-3 PUFA. The aim of this study was to employ high throughput lipidomic techniques to evaluate the effect of diets high or low in *n*-3 PUFA content on the fatty acid composition of various lipids in the plasma and liver of C57BL/6 mice. Our findings reveal for the first time that diets high in *n*-3 PUFA caused enrichment of *n*-3 PUFA in PC, LPC, cholesteryl esters (CE) and FFA in the plasma and liver of C57BL/6 mice, which will likely have important physiological roles. 

## Materials and Methods

### Ethics statement

All experimental procedures were approved by Memorial University Animal Care Committee in accordance with the principles and guidelines of Canadian Council on Animal Care (approval no: 10-09-SK).

### Animals and diets

Seven week old male and female C57BL/6 mice were purchased from Charles River Laboratories (MA, USA), and were housed in separate cages under controlled temperature (21± 1°C) and humidity (35 ± 5%) conditions with a 12-hour light/12-hour dark period cycle. Mice were kept on standard rodent chow pellets (Prolab RMH 3000) purchased from PMI nutrition (MO, USA) for one week acclimatization period. After this period, female mice were randomly divided into two groups. Each group was fed either of the two experimental diets that differed only in their *n*-3 PUFA composition, and designated as “high *n*-3” and “low *n*-3” diets for two weeks before mating. The experimental diets were made from a base semi-synthetic diet (MP Biomedicals, OH, USA), which allows for the control of fat level at 20% w/w. Menhaden oil was obtained from Sigma-Aldrich (MO, USA); lard, safflower oil and extra-virgin olive oil were used to prepare two different oil mixtures containing 10% (high *n*-3) and 2% (low *n*-3) *n*-3 PUFA of the total fat, giving an *n*-6 to *n*-3 PUFA ratio of 5:1 and 30:1 respectively. The fatty acid composition of the diets was analyzed by gas chromatography as per our previous publication [38] and is given in Table 1. Females were continued on the experimental diets throughout gestation, lactation, and until weaning. Just before weaning, breast milk samples were collected from the mothers, to ascertain that the dietary essential fatty acid is reflected in the breast milk. There was a positive correlation between dietary and breast milk fatty acids composition (data not shown). At weaning, male offspring (*n*=6) were continued on their mothers’ designated diet for 16 weeks (i.e. offspring obtained from mother’s fed a high *n*-3 PUFA diet continued on high *n*-3 PUFA diet). Only male offspring were used in this study in order to prevent hormonal interference. Female sex hormones have been shown to affect tissue plasma and tissue n-3 PUFA content [39]; and it is well known that there is gender specificity in lipid metabolism, possibly due to the effect of hormonal differences [39-42]. Animals were provided with water and fresh food *ad libitum*, every other day. Body weights were recorded once a week, and food intake was recorded every other day. No significant differences were observed in both body weight and food intake (*Table S1 *in File *S1*). At 16 weeks, male offspring were fasted overnight and sacrificed using isoflurane. Blood was collected by cardiac puncture in tubes containing EDTA (4.5 mM, pH 7.4), and plasma was separated immediately. Tissues were removed and weighed at the time of sacrifice, snap frozen in liquid nitrogen and stored at -80°C until further analyses.

**Table 1:** Fatty acid composition of the experimental diets.

**Fatty Acid**	**High *n*-3**	**Low *n*-3**
C14:0	1.26	0.11
C16:0	8.71	6.32
C18:0	2.67	5.35
**ΣSFA**	12.64	11.77
C16:1n7	2.41	0.36
C18:1n9 + C18:1n7	25.14	27.82
C20:1n9	0.61	ND
**ΣMUFA**	28.16	28.18
C18:2n6	47.86	57.73
C20:4n6	0.23	0.11
C18:3n6	0.10	0.04
C22:4n6	0.54	0.09
**ΣOmega-6**	48.90	57.92
C18:3n3	0.78	0.55
C20:5n3	3.64	0.31
C22:6n3	3.19	0.39
C18:4n3	0.87	0.15
C22:5n3	0.63	0.46
C20:4n3	0.66	0.08
**ΣOmega-3**	9.76	1.93
**ΣPUFA**	59.38	60.00
**ΣOmega-6/Omega-3**	5.00	30.01

Lipids were extracted from the diets and the fatty acid composition was determined by gas chromatography. Values are given as % area of each fatty acid peak. Abbreviations: ND= Not detected, ΣSFA= sum of saturated fatty acids, ΣMUFA= sum of monounsaturated fatty acids, Σ PUFA= sum of polyunsaturated fatty acids, ΣOmega-6= sum of omega-6 fatty acids, ΣOmega-3= sum of omega-3 fatty acid.

### Lipidomic analysis

#### Lipid extraction, standards, and solvents

Plasma or liver samples were flash frozen at the temperature of liquid nitrogen at collection. 10 µl plasma was directly extracted into organic solvent, while 100 mg liver tissue was pulverized and homogenized and then immediately lipids were extracted into organic solvent. Lipid extraction into organic solvent was performed using the Bligh-Dyer method [43] with high performance liquid chromatography-mass spectrometry grade solvents in the presence of non-naturally occurring internal lipid standards. The standards used were Δ9-trans-triheptadecenoin (tri-17:1 TG), 1, 2-diarachidoyl-*sn*-glycero-3-phosphocholine (di-20:0 PC), N-heptadecanoyl-D-*erythro*-sphingosylphosphorylcholine (17:0 sphingomyelin), 1-heptadecanoyl-2-hydroxy-*sn*-glycero-3-phosphocholine (17:0 LPC), 1, 2-dimyristoyl-sn-glycero-3-phosphoethanolamine (di-14:0 PE), cholesteryl heptadecanoate (17:0 CE), and N-hepadecanoyl-D-*erythro*-sphingosine (17:0 ceramide). The extracted lipids in the chloroform phase were dried down under gentle stream of N_2_ gas, and re-suspended in 500 µl of chloroform. 50 µl aliquot of the suspension was then mixed with 200µl methanol and 2µl of 10 mM methanolic NaOH and injected into a Thermo Fisher TSQ Quantum Ultra tandem electrospray ionization mass spectrometry (ESI-MS) system for lipid analyses. High pressure liquid chromatography-mass spectrometry grade methanol and chloroform were used for all extractions; these solvents were purchased from Burdick and Jackson (NJ, USA).

#### Electrospray ionization-mass spectrometry (ESI-MS)

Samples were analysed using direct-infusion ESI-MS in positive or negative ion mode using a Thermo Fisher TSQ Quantum Ultra system with XCalibur data acquisition software [44]. The tune parameters for sample analyses were optimized and set as follows: spray voltage = 3500 V, flow rate = 3 µl/min, ion sweep gas pressure = 0.2 (arbitrary units), sheath gas = 12 (arbitrary units), auxiliary gas pressure = 6 (arbitrary units), and capillary temperature = 270°C. The collision energies for the analyses of PC, LPC, and sphingomyelins (SM) were set at 28 eV. The collision energies for analyses of cholesteryl esters (CE) was set at 25 eV, and for ceramide (CER) was set at 32 eV. CEs were detected in positive ion mode by scanning for neutral loss (NL) of cholestadane (*m/z* 368.5). Sodiated species of SM, PC, and LPC were detected in positive ion mode by scanning for the NL of choline (*m/z* 59.1). FFA and PE were identified in negative ion mode by survey scan for [M-H] ^-^ between *m/z* 200 and 900. Sodiated species of TG were identified in positive ion mode by survey scan for [M+Na]^+^ between *m/z* 800 and 1000. All data analyzed were corrected for ^13^C isotope effects as described by Han et al. [44].

### Statistical analysis

Data were analysed using GraphPad Prism software (version 5.0). Statistical significance for differences between groups was determined by unpaired t-test. Results are expressed as mean ± standard deviation (SD). Differences were considered to be statistically significant if the associated *P* value was < 0.05. 

## Results

### PC fatty acyl composition of mice fed high and low n-3 PUFA

**Figure 1:**
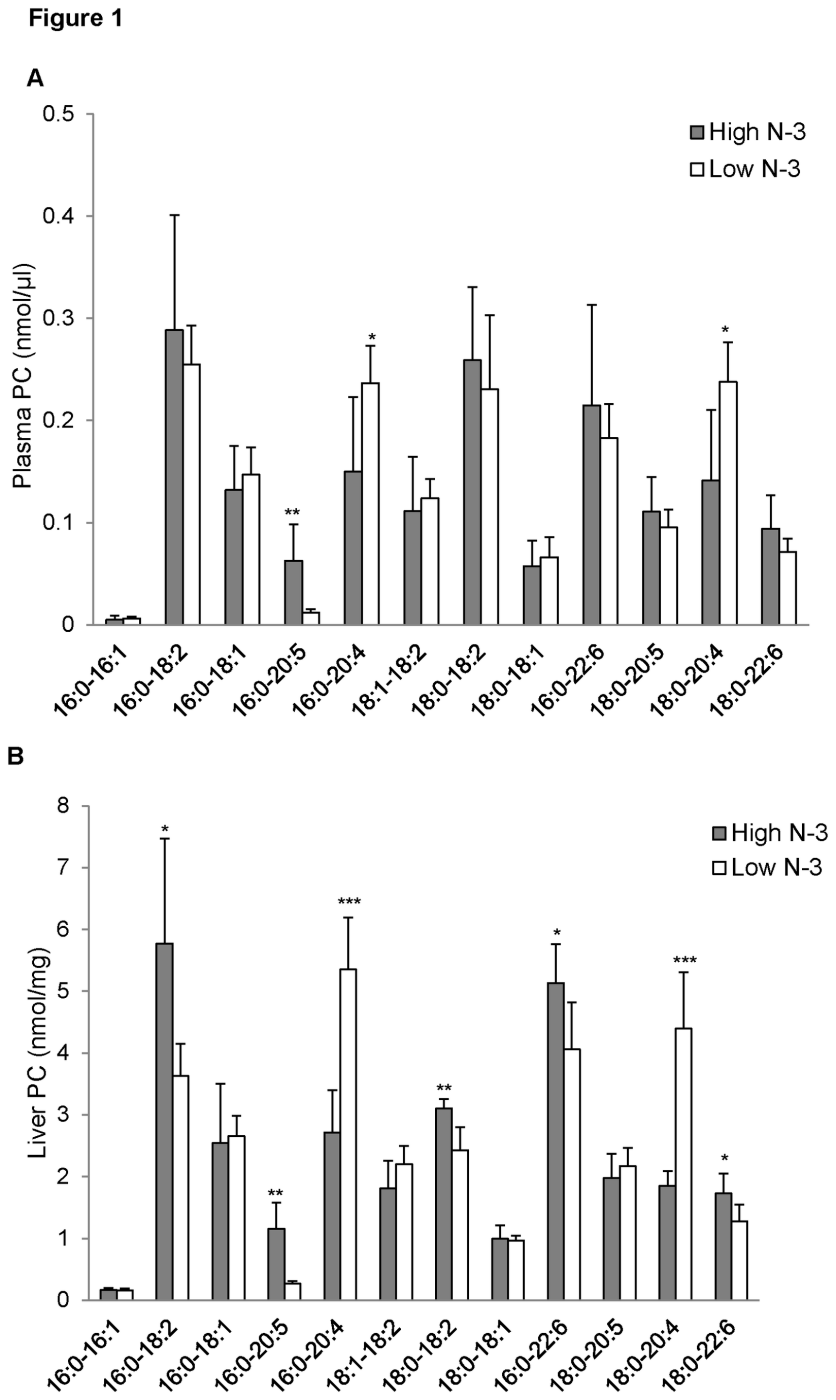
Phosphatidylcholine (PC) fatty acyl composition of mice fed high and low *n*-3 PUFA enriched diets. Plasma (A) and liver (B) PC species were quantified by measuring the sodiated adducts of PC using ESI-MS in positive ion mode by NL scanning for *m/z* 59.1, as described in the “Methods”. Data were corrected for ^13^C isotopic effects. Data are presented as mean (*n*=6) ± SD and *P*-values calculated using unpaired t-test. *P < 0.05; **P < 0.01; ***P < 0.001.

There were no significant differences in the total concentrations of phospholipids in both plasma and liver between high and low *n*-3 PUFA fed mice (*Table S2 *in File *S1*). However, there was a significantly higher concentration of 16:0-20:5 PC in the plasma (*p* < 0.01; Figure 1A) and liver (*p* < 0.05; Figure 1B) of the high *n*-3 PUFA group compared to the low *n*-3 PUFA group. Interestingly, 20:4 containing PC (16:0-20:4 and 18:0-20:4) increased significantly in the plasma of the low *n*-3 PUFA group compared to the high *n*-3 PUFA group (*p* < 0.05; Figure 1A). A similar effect was observed in the liver, with 16:0-20:4 and 18:0-20:4 showing a significant increase in the low *n*-3 PUFA group (*p* < 0.001; Figure 1B). This signifies a higher incorporation of EPA and a lower incorporation of AA into plasma PC in the high *n*-3 PUFA group. The high *n*-3 PUFA group also showed higher incorporation of DHA into hepatic PC compared to the low *n*-3 PUFA group (*p* < 0.05; Figure 1B); however, there were no differences in plasma DHA containing PC in both groups (Figure 1A). 

### FFA of mice fed high and low n-3 PUFA

**Figure 2:**
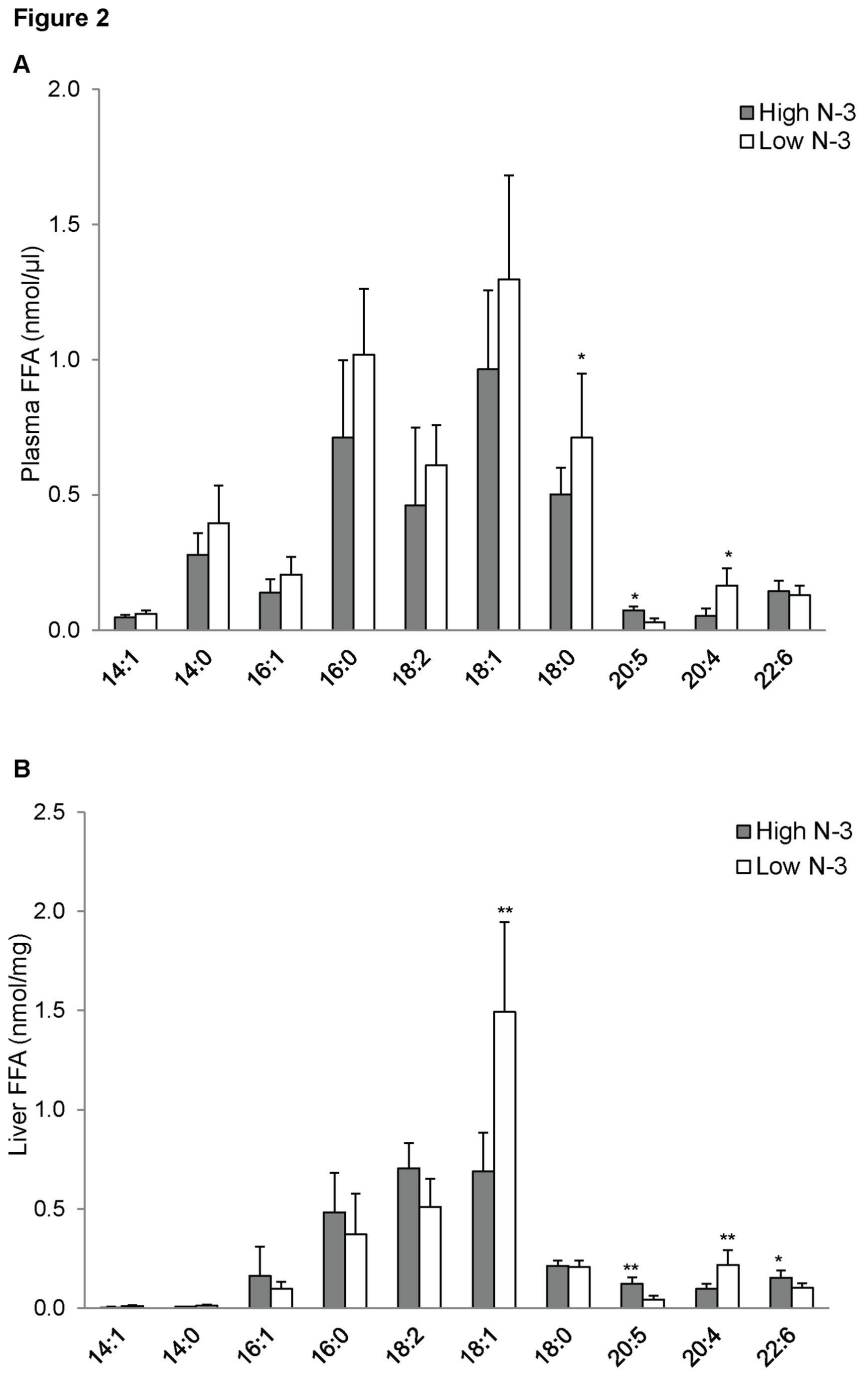
Free fatty acid (FFA) composition of mice fed high and low *n*-3 PUFA enriched diets. Plasma (A) and liver (B) FFA composition was quantified by measuring [M-H]^-^ using ESI-MS in negative ion mode between *m/z* 200 and 900. Data were corrected for ^13^C isotopic effects. Data are presented as mean (*n*=6) ± SD and *P*-values calculated using unpaired t-test. *P < 0.05; **P < 0.01; ***P < 0.001.

There was a significant accretion of AA in the plasma of the low *n*-3 PUFA group compared to the high *n*-3 PUFA group (*p* < 0.05; Figure 2A). The plasma concentration of EPA was considerably higher in the high *n*-3 PUFA group compared to the low *n*-3 PUFA group (*p* < 0.05; Figure 2A). However, no significant difference was observed in the plasma concentration of DHA in both groups. Livers of the mice fed a low *n*-3 PUFA diet were significantly enriched with 18:1 and 20:4 FFA (*p* < 0.01; Figure 2B) compared to the high *n*-3 PUFA group. Similarly, the liver concentrations of EPA (*p* < 0.01; Figure 2B) and DHA (*p* < 0.05; Figure 2B) were significantly higher in the high *n*-3 PUFA group compared to the low *n*-3 PUFA group. 

### LPC fatty acyl composition of mice fed high and low n-3 PUFA

**Figure 3:**
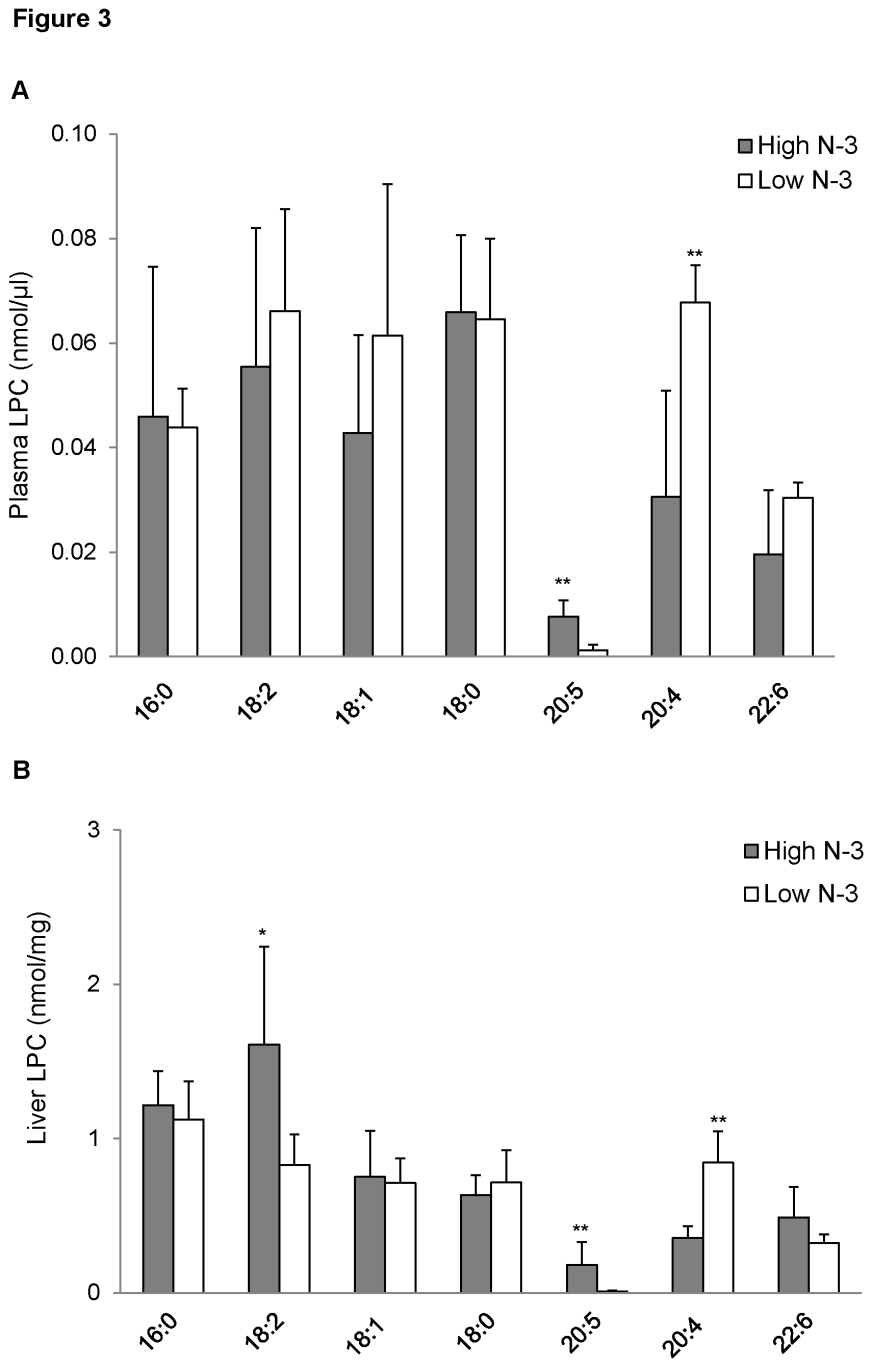
Lysophosphatidylcholine (LPC) fatty acyl composition of mice fed high and low *n*-3 PUFA enriched diets. Plasma (A) and liver (B) LPC species were quantified by measuring the sodiated adducts of PC using ESI-MS in positive ion mode by NL scanning for *m/z* 59.1, as described in the “Methods”. Data were corrected for ^13^C isotopic effects. Data are presented as mean (*n*=6) ± SD and *P*-values calculated using unpaired t-test. *P < 0.05; **P < 0.01; ***P < 0.001.

Similar to the PC data, the concentration of 20:5 LPC was significantly higher in the high *n*-3 PUFA group compared to the low *n*-3 PUFA group, in both plasma (*p* < 0.01; Figure 3A) and liver (*p* < 0.01; Figure 3B). However, there was a significant accretion of 20:4 LPC in the plasma (*p* < 0.01; Figure 3A) and liver (*p* < 0.05; Figure 3B) of the low *n*-3 PUFA group compared to the high *n*-3 PUFA group. Irrespective of the differences observed in LPC, there was no significant difference in the total concentration of LPC in both groups (*Table S2 *in File *S1*).

### PE fatty acyl composition of mice fed high and low n-3 PUFA

**Figure 4:**
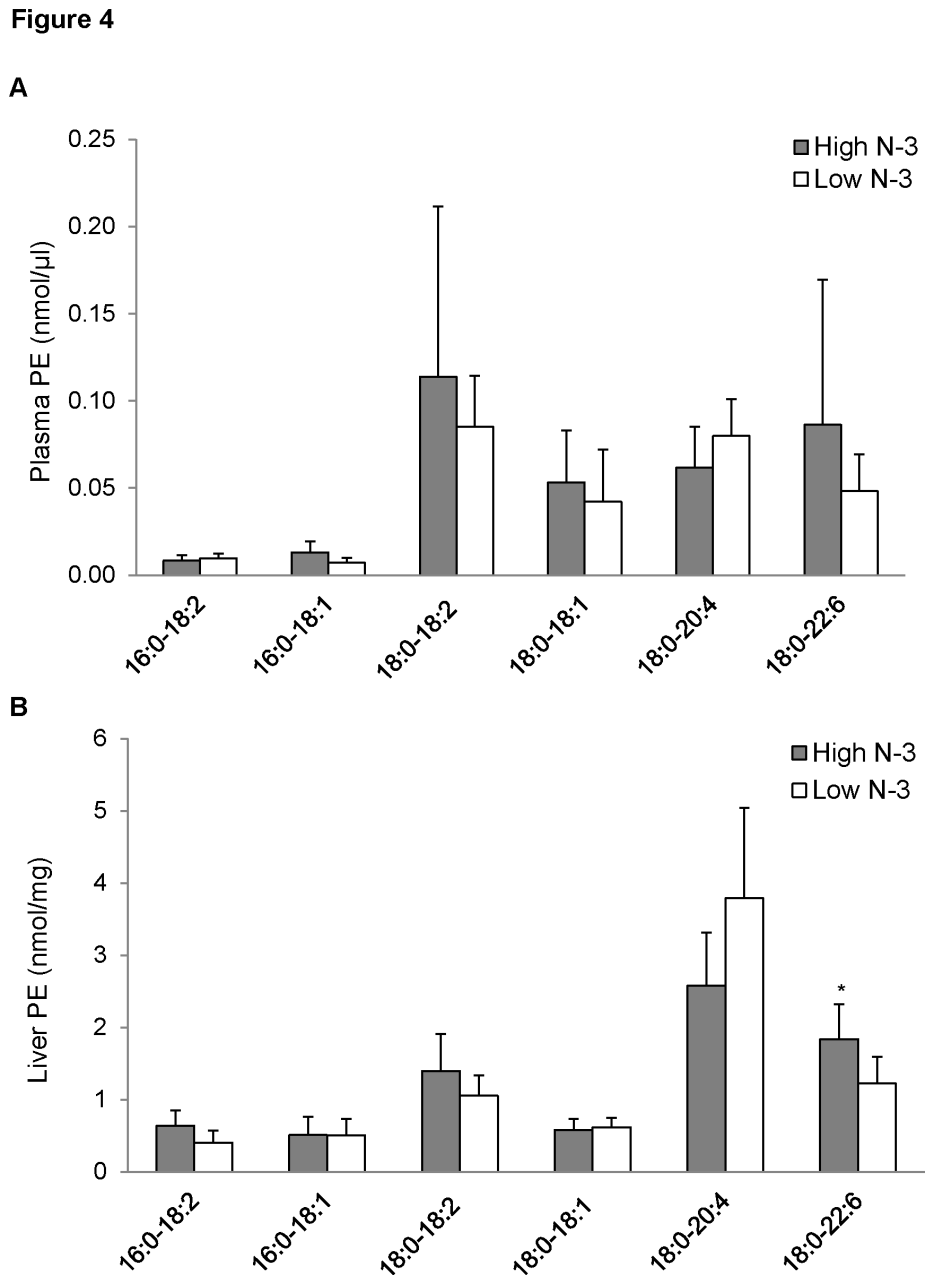
Phosphatidylethanolamine (PE) fatty acyl composition of mice fed high and low *n*-3 PUFA enriched diets. Plasma (A) and liver (B) levels of PE species were quantified by measuring [M-H]^-^ using ESI-MS in negative ion mode between *m/z* 200 and 900. Data were corrected for ^13^C isotopic effects. Data are presented as mean (*n*=6) ± SD and *P*-values calculated using unpaired t-test. *P < 0.05; **P < 0.01; ***P < 0.001.

No significant differences were observed in the fatty acyl species of PE in the plasma (Figure 4A) of the high *n*-3 PUFA and low *n*-3 PUFA groups. The liver showed no differences amongst PE species containing EPA; however, there was a significant increase of hepatic 18:0-22:6 PE in the high *n*-3 PUFA group compared to the low *n*-3 PUFA group (*p* < 0.05; Figure 4B).

### Sphingolipid and ceramide fatty acyl composition of mice fed high and low n-3 PUFA

**Figure 5:**
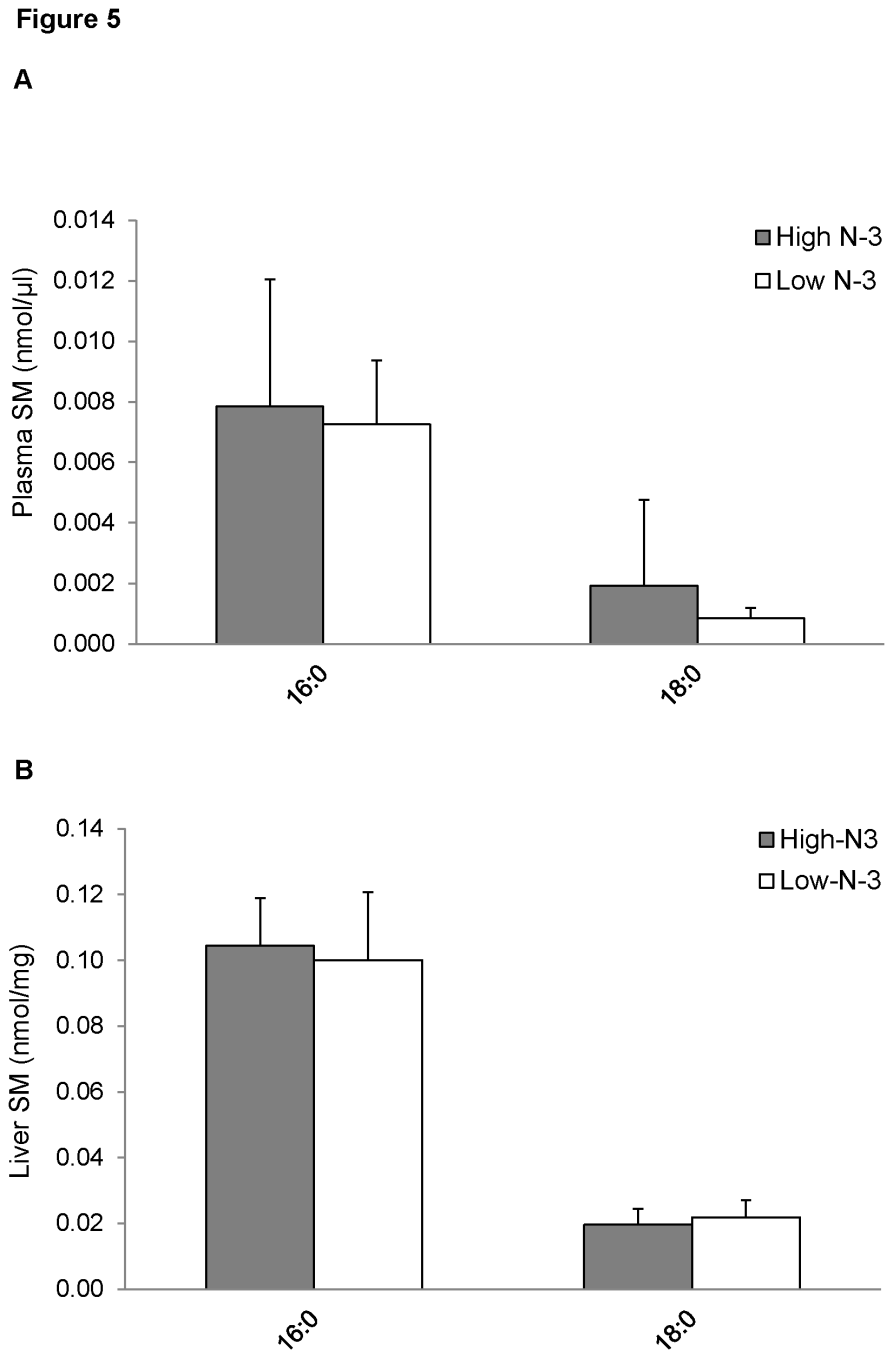
Sphingomyelin (SM) fatty acyl composition of mice fed high and low *n*-3 PUFA enriched diets. Plasma (A) and liver (B) levels of SM species were quantified by measuring the sodiated adducts of PC using ESI-MS in positive ion mode by NL scanning for *m/z* 59.1, as described in the “Methods”. Data were corrected for ^13^C isotopic effects. Data are presented as mean (*n*=6) ± SD and *P*-values calculated using unpaired t-test. *P < 0.05; **P < 0.01; ***P < 0.001.

**Figure 6:**
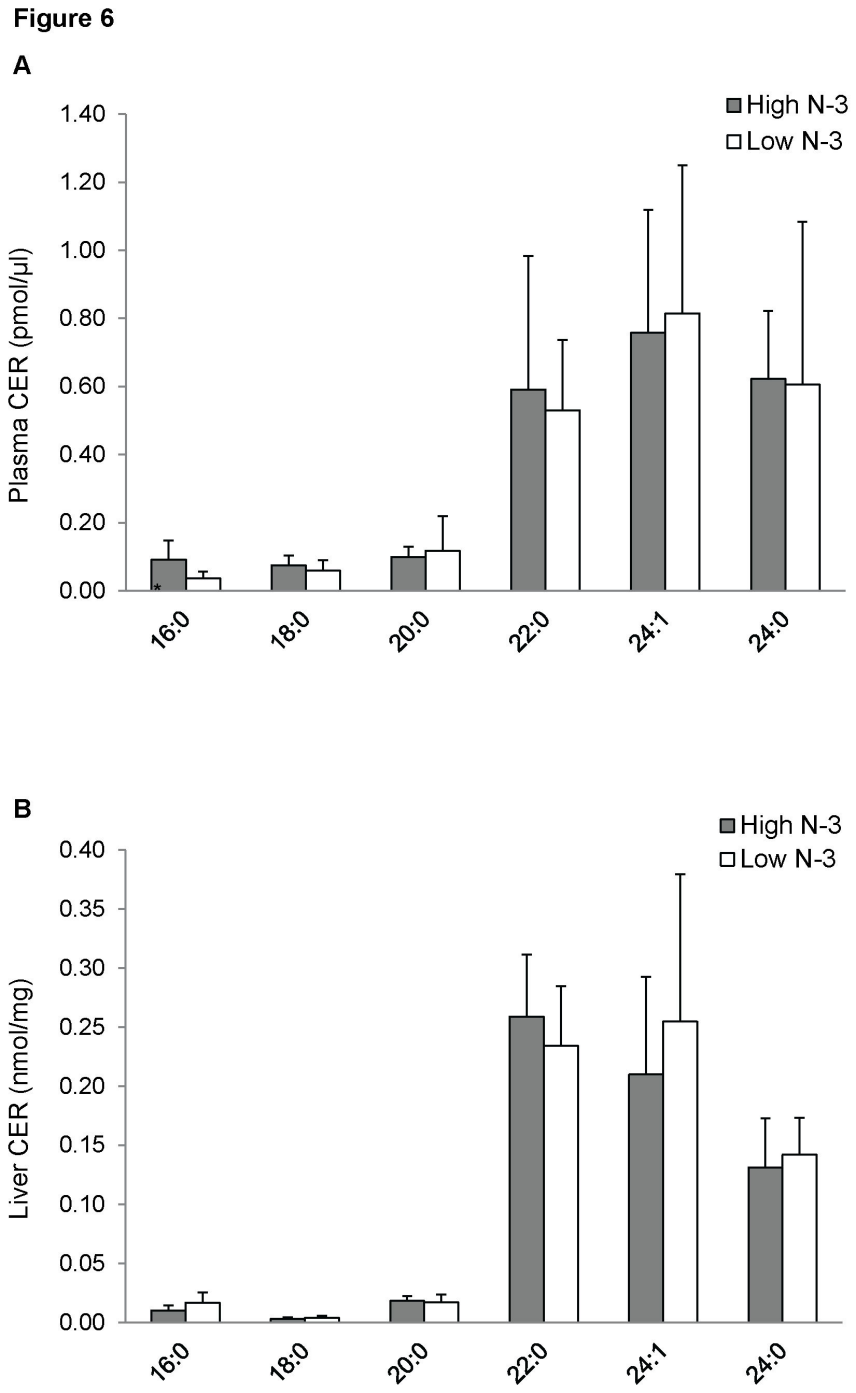
Ceramide (CER) fatty acyl composition of mice fed high and low *n*-3 PUFA enriched diets. Plasma (A) and liver (B) CER species were quantified using ESI-MS in negative ion mode by NL scanning for *m/z* 256.2. Data were corrected for ^13^C isotope effects. Data are presented as mean (*n*=6) ± SD and *P*-values calculated using unpaired t-test. *P < 0.05; **P < 0.01; ***P < 0.001.

Treatment with high *n*-3 PUFA did not significantly modify the fatty acyl species of SM in the plasma (Figure 5A) and liver (Figure 5B) of mice fed diet high in *n*-3 PUFA. Plasma of mice from high *n*-3 PUFA group showed an increase in 16:0 CER compared to the low *n*-3 PUFA group (*p* < 0.05 Figure 6A); however, there was no effect of diet on different fatty acyl species of CER in the liver (Figure 6B). 

### CE and TG fatty acyl composition of mice fed high and low n-3 PUFA

**Figure 7:**
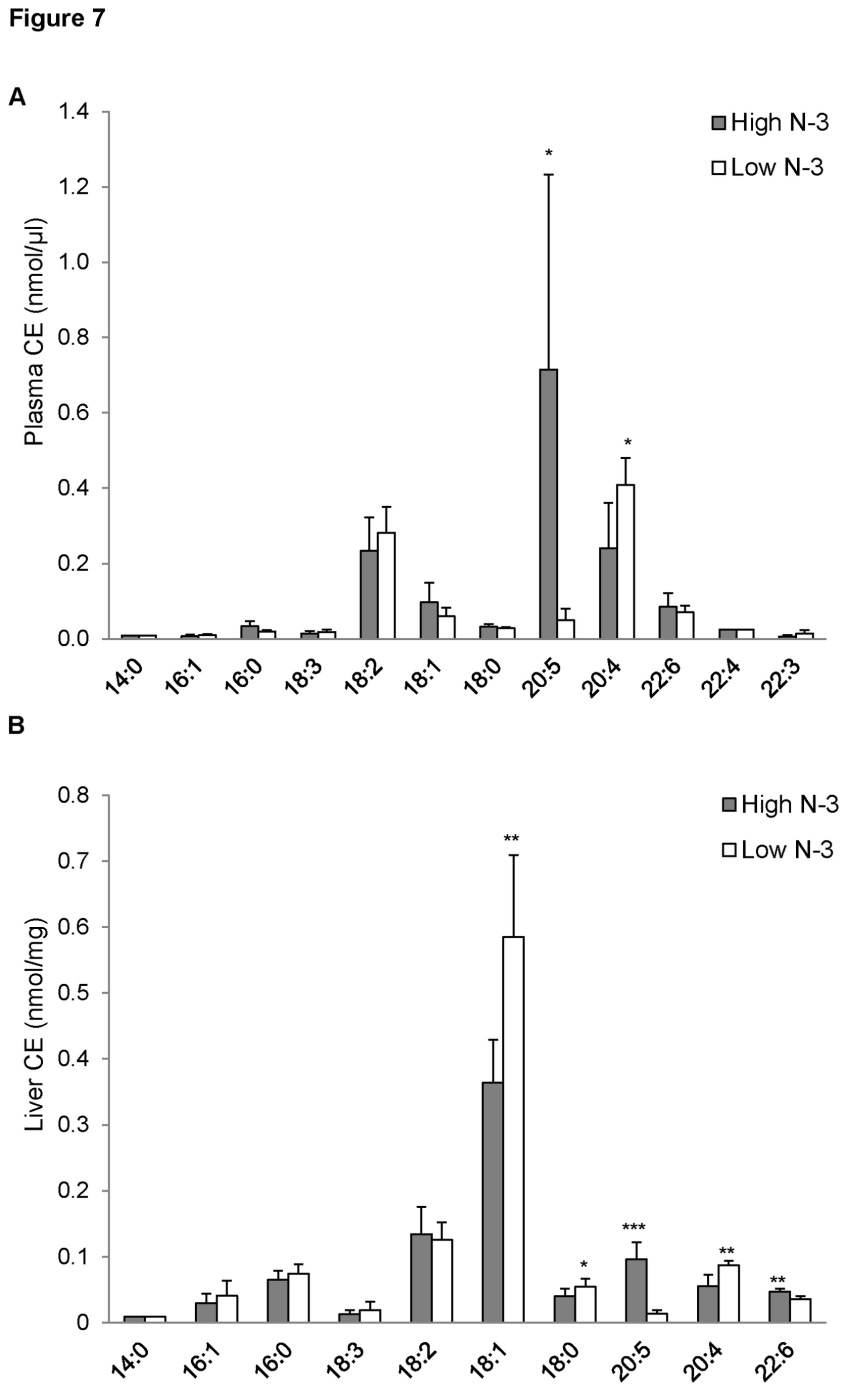
Cholesteryl ester (CE) fatty acyl composition of mice fed high and low *n*-3 PUFA enriched diets. Plasma (A) and liver (B) CE species were quantified using ESI-MS in positive ion mode by NL scanning of *m/z* 368.5 as described in the “Methods”. Data were corrected for ^13^C isotope effects. Data are presented as mean (*n*=6) ± SD and *P*-values calculated using unpaired t-test. *P < 0.05; **P < 0.01; ***P < 0.001.

The high *n*-3 PUFA group showed a significant increase in plasma 20:5 CE compared to the low *n*-3 PUFA group (*p* < 0.05; Figure 7A). Conversely, there was a high accumulation of plasma 20:4 CE in the low *n*-3 PUFA group compared to the high *n*-3 PUFA group (*p* < 0.05; Figure 7A). The liver of the high *n*-3 PUFA group was enriched in 20:5 CE (*p* < 0.001; Figure 7B) and 22:6 CE (*p* < 0.01; Figure 7B) compared to the low *n*-3 PUFA group. The liver concentration of 20:4 CE was significantly higher in the low *n*-3 PUFA group (*p* < 0.01; Figure 7B) compared to the high *n*-3 PUFA group. 

**Figure 8:**
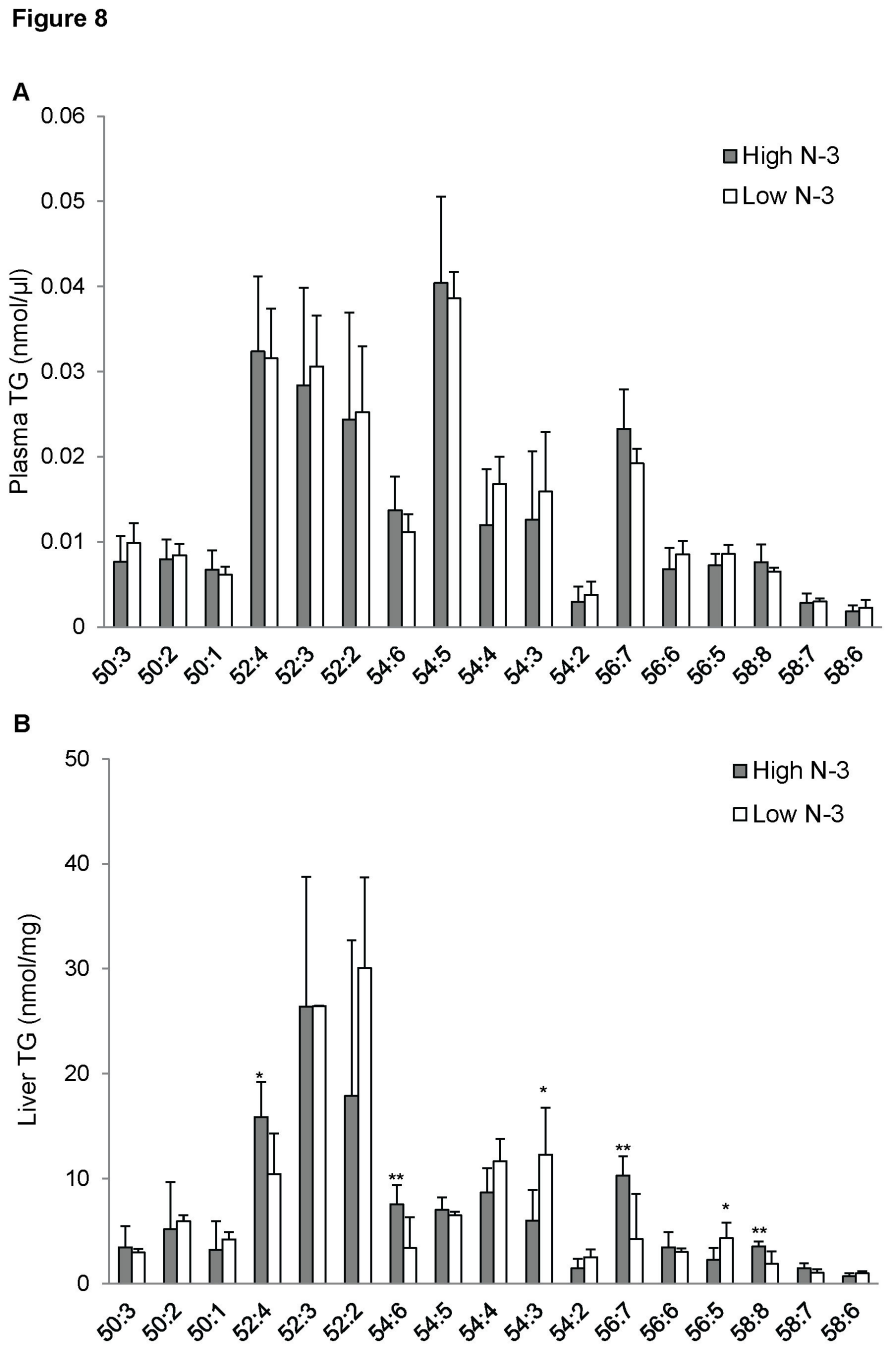
Triglyceride (TG) fatty acyl composition of mice fed high and low *n*-3 PUFA enriched diets. Plasma (A) and liver (B) TG species were quantified by measuring [M+Na]^+^ using ESI-MS in positive ion mode between *m/z* 800 and 1000 described in the “Methods”. Data were corrected for ^13^C isotope effects. Data are presented as mean (*n*=6) ± SD and *P*-values calculated using unpaired t-test. *P < 0.05; **P < 0.01; ***P < 0.001.

No differences were observed in the concentrations of the different fatty acyl species of TG found in the plasma in both experimental groups (Figure 8A). Nevertheless, the liver showed a distinctive difference in the concentration of fatty acyl species profile for TG. The concentrations of 54:6, 56:7, and 58:8 TGs were significantly greater in the high *n*-3 PUFA group compared to the low *n*-3 PUFA group (*p* < 0.01; Figure 8B). The low *n*-3 PUFA group showed a significantly higher 54:3 and 56:5 TG species compared to the high *n*-3 PUFA group (*p* < 0.05; Figure 8B). 

## Discussion

*N*-3 PUFA have been shown to reduce plasma TG [8,45], prevent atherosclerosis [46], and alleviate inflammation [47]. It has been proposed that the beneficial effects of *n*-3 PUFA are mediated through the actions of bioactive lipid components; however which bioactive lipids are metabolically active, and their mechanisms of action are still not clear. We performed lipidomic analyses on plasma and liver samples of high and low *n*-3 PUFA fed male C57BL/6 mice. There was an evident distinction in the fatty acid profiles of different lipids present in the plasma and liver of the two dietary groups. Feeding a high *n*-3 PUFA diet led to a marked reduction of AA containing PC, with a concomitant increase of EPA containing PC. Although plasma samples from mice fed a high or low *n*-3 PUFA diet showed no difference in DHA containing PC, there was a trend towards an increase in the high *n*-3 PUFA group. Browning et al. reported that habitual supplementation of fish oil led to an increased incorporation of EPA in plasma PC [48]; however, they did observe a significant incorporation of DHA into PC after dietary supplements of DHA [48]. A plausible explanation for the disparity in our findings is that DHA containing PC is rapidly converted to LPC for delivery to extrahepatic tissues. The liver PC fatty acyl composition is consistent with changes in plasma PC, where high dietary *n*-3 PUFA increased the concentrations of hepatic EPA and DHA species of PC. On the contrary, there was a significant reduction of hepatic AA containing PC in mice fed high *n*-3 PUFA diet. PC is an important phospholipid involved in lipid metabolism. PC has been shown to play a major role in cellular proliferation, degeneration, and membrane fluidity and functions [13]. The physiological properties of PC are heavily dependent on their fatty acid composition and *n*-3 PUFA has been shown to be preferentially incorporated into PC [48]. PC from marine sources are rich in *n*-3 PUFA and have been shown to significantly reduce markers of inflammation [49], by the inhibition of TNF-α induced activity of NF-κB [50]. In addition to their anti-inflammatory effects, *n*-3 PUFA rich PC are also known to possess lipid lowering effects [51-53]. 

Plasma PC is rapidly metabolised by phospholipase A_2_ to produce FFA and LPC. These bioactive metabolites of PC can also elicit beneficial effects depending on the type, chain length, and degree of unsaturation of their fatty acids. Feeding diets enriched in *n*-3 PUFA caused an increase in the plasma concentration of EPA and a decrease in plasma concentration of AA. PC is an important source of FFA in plasma [48]. We found a high concentration of EPA containing PC in the high *n*-3 PUFA group; it is thus reasonable to assume that the high plasma concentration of EPA found in the high *n*-3 PUFA fed mice is partly a product of enzyme hydrolysis of EPA rich PC. This is in line with previous findings that support the incorporation of *n*-3 PUFA into the plasma pool when given as dietary supplements [48,54]. We observed that the dietary supplementation with high *n*-3 PUFA caused an increase in the concentrations of hepatic EPA and DHA, and a decline in the hepatic concentration of AA. Our observation is in line with the study of Lamaziere et al., who reported an increase in hepatic EPA and DHA after rats were administered fish oil for 30 days [55]. In addition to the aforementioned health benefits of *n*-3 PUFA, high hepatic *n*-3 PUFA are also involved in inhibiting lipogenesis thereby preventing the development of non-alcoholic fatty liver disease (NAFLD) [55]. Mechanistically, *n*-3 PUFA has been shown to regulate lipogenesis by inhibiting LXR and SREPB-1c [56,57]. Chronic low-grade inflammation underlies the pathology of most metabolic disorders, and free *n*-3 PUFA has been shown to possess potent anti-inflammatory properties [58]. *N*-3 PUFA alleviates inflammation by directly regulating transcription factors involved in inflammation [59-61] and indirectly by producing series-3 and series-5 eicosanoids [62,63]. In addition to their inflammation resolving properties, free unesterified *n*-3 PUFA have also been shown to improve symptoms of dyslipidaemia [9-11]. 

Another important bioactive product of enzyme hydrolysis of PC is LPC. We found that feeding a diet high in *n*-3 PUFA considerably increased plasma circulating levels of 20:5 LPC, and drastically reduced plasma concentration of 20:4 LPC. Similar to our plasma data, we found a higher concentration of hepatic 20:5 LPC and a low concentration of hepatic 20:4 LPC in the high *n*-3 group. Interestingly, there was no significant difference in hepatic 22:6 LPC, although there was a trend towards an increase in the high n-3 PUFA group. Ottestad et al. recently reported a similar finding where fish oil supplementation to healthy humans significantly increased plasma 20:5 LPC and 22:6 LPC [64]. Another study by Block et al. also found that fish oil supplementation increased EPA and DHA containing LPC [65]. However, we only found a change in plasma EPA containing LPC, while there was no difference in DHA containing LPC between the two groups. LPC has been suggested as the major carrier of DHA to the brain tissues [66]; tracer studies revealed that labelled LPC injected into the blood of rat disappeared within 20 s and were recovered in different organs including the brain [17]. It would thus be logical to believe that the DHA containing LPC was rapidly cleared from circulation. 

Studies have controversially linked LPC with the development of atherosclerosis [25-27]. This is possibly due to their association with oxidized LDL, and promotion of inflammation [67,68] by generating reactive oxygen species and nitric oxides in different types of cells [69,70]. However, the studies that linked LPC with the pathogenesis of obesity, diabetes, and rheumatoid arthritis have reported an increase in saturated LPC [30]. Of paramount importance to the biological functions of LPC are acyl length and degree of saturation of their fatty acids [65]. LPC rich in *n*-3 PUFA have been shown to possess beneficial properties. *N*-3 PUFA species of LPC were found to reduce inflammation [71,72], and LPC containing DHA at *sn*-1 position exhibits significantly higher anti-inflammatory properties compared to LPC with either linoleic or arachidonic acid [31,73,74]. It has been proposed that LPC containing *n*-3 PUFA exhibit their anti-inflammatory properties through hydrolytic cleavage of the *n*-3 PUFA moiety, or oxygenation by 15-lipooxygenase (15-LOX) to produce inflammation resolving lipids such as 1-(15-hydroperoxyeicosatetraenoyl)-LPC and 1-(17- hydroperoxydocosahexaenoyl)-LPC [74]. Most of the available data on the health benefits of *n*-3 PUFA rich LPC were shown using LPC containing *n*-3 PUFA at *sn*-1 position. We have not investigated the position of the acyl group of our LPC, however, it is known that there is rapid isomerization of acyl group from *sn*-2 to a more stable *sn*-1 position in LPC [75]. It has been reported that after separation of blood from plasma, 90% of the unsaturated fatty acids were found at *sn*-1 position in LPC [24]. We can therefore speculate that a significant percentage of the plasma 20:5 LPC contain EPA at *sn*-1 position. 

Also noteworthy is the fact that, despite the changes in fatty acyl species of PC and LPC in response to diet, there was no difference in the total concentrations of PC and LPC between the two experimental groups. A similar observation was found by Ottestad et al., where they found no difference in total concentrations of PC and LPC despite the apparent changes in individual fatty acyl species in response to fish oil supplementation [64]. This interesting observation suggests that the functional properties of *n*-3 PUFA involve remodelling and improving the quality of bioactive lipid mediators without affecting their concentrations.

A diet rich in *n*-3 PUFA had no effect on different fatty acyl species of plasma TG, confirming preferential incorporation of *n*-3 PUFA into PC. However, the high *n*-3 PUFA diet led to an increase in plasma and liver concentrations of *n*-3 PUFA CE, and a decrease in 20:4 CE. CE is less polar than free cholesterol and it functions as an inert storage molecule. The high incorporation of *n*-3 PUFA in CE could be explained as a response to the high *n*-3 PUFA in the diet. This would simply indicate that *n*-3 PUFA are stored and will be later released for other physiological functions. A cross sectional study has reported a positive correlation between dietary PUFA and CE [76].

Although we have shown that there is high incorporation of *n*-3 PUFA in the liver and plasma PC and LPC of mice fed high *n*-3 diet, it was also imperative to ascertain the effect of high *n*-3 PUFA diet on PE, which is also abundant in the membranes. There was no difference in plasma PE between the two dietary groups; the only difference detected in the liver was an increase in 18:0-22:6 PE in the high *n*-3 group. Our findings confirm that fish oil supplementation leads to a preferential incorporation of *n*-3 PUFA into PC and LPC compared to other phospholipids [64]. 

Sphingolipids, such as CER and SM, are important signalling molecules. CER is a sphingolipid linked with inflammation and the pathogenesis of cardiovascular diseases (CVD) [77,78]. There are also speculations on the involvement of CER in insulin signalling, although available information is scanty [79,80]. We found no difference in total concentration of CER in plasma and liver. Our findings are similar to Ottestad et al., who found that fish oil supplement had no effect on plasma concentration of CER. Interestingly, we found an increase in 16:0 CER in the high *n*-3 PUFA group. The functional roles of this CER species are currently unknown and needs to be explored. There was no difference in plasma and liver SM concentrations between the two groups. The physiological functions of SM have not been extensively studied. However, it is known that SM are involved in the formation of specialized membrane microdomains known as lipid rafts involved in signalling. 

In conclusion, our findings have shown that diets high in *n*-3 PUFA alter plasma and liver lipidomic profile of the offspring. We found that *n*-3 PUFA is preferentially incorporated into PC and LPC, and despite the changes in lipidomic profile, the total concentrations of these lipids were not altered. Additionally, we found that dietary *n*-3 PUFA is capable of remodelling the fatty acyl moieties of PC, LPC, and CE, which may have important physiological implications, and needs to be further investigated. Future studies will be undertaken to investigate the mechanism(s) by which *n*-3 PUFA remodelled bioactive lipids regulate metabolic pathways. 

## Supporting Information

File S1 **Table S1, Body weight at sacrifice, and average weekly food intake of mice fed high and low n-3 PUFA diets; Table S2, Total concentrations of plasma and liver phospholipids of mice fed high and low n-3 PUFA diets.**
(DOCX)
