# Supplementary material for: Dietary Omega-3 Polyunsaturated Fatty Acids Alter the Fatty Acid Composition of Hepatic and Plasma Bioactive Lipids in C57BL/6 Mice: A Lipidomic Approach
Source: PLoS One. 2013 Nov 21;8(11):e82399. doi: 10.1371/journal.pone.0082399 (PMC3836780; doi:10.1371/journal.pone.0082399)
Supplement: File S1 — Table S1, Body weight at sacrifice, and average weekly food intake of mice fed high and low n-3 PUFA diets; Table S2, Total concentrations of plasma and liver phospholipids of mice fed high and low n-3 PUFA diets. (DOCX) [file pone.0082399.s001.docx]

**Table S1. Body weight at sacrifice, and average weekly food intake of mice fed high and low *n*-3 PUFA diets**

|  | **High *n*-3** | **Low *n*-3** |
| --- | --- | --- |
| **Body Weight (g)** | 29.77± 2.33 | 28.18 ± 3.60 |
| **Food Intake (g/week)** | 22.94 ± 0.70 | 22.27 ± 0.98 |

Values are expressed as means ± SD, *n*=6. Data were analyzed using unpaired t-test. PUFA= Polyunsaturated fatty acids

**Table S2. Total concentrations of plasma and liver phospholipids of mice fed high and low *n*-3 PUFA diets**

|  |  |  |  |  |
| --- | --- | --- | --- | --- |
|  | **PLASMA**  **(nmol/µl)** | | **LIVER**  **(nmol/mg)** | |
|  | **High *n*-3** | **Low *n*-3** | **High *n*-3** | **Low *n*-3** |
| **PC** | 1.6 ± 0.51 | 1.7 ± 0.18 | 28.9 ± 3.00 | 29.6 ± 3.82 |
| **LPC** | 0.3 ± 0.05 | 0.3 ± 0.06 | 4.3 ± 0.92 | 4.3 ± 1.11 |
| **PE** | 0.3 ± 0.22 | 0.3 ± 0.08 | 7. 6 ± 2.19 | 7.6 ± 2.30 |
| **SM** | 0.01 ±0 .005 | 0.01 ± 0.002 | 0.12 ± 0.01 | 0.12 ± 0.03 |
| **CER*** | 2.7 ± 0.90 | 2.7 ± 1.56 | 0.6 ± 0.15 | 0.7 ± 0.15 |

Total concentrations of phospholipids were quantified using ESI-MS. Values are expressed as means ± SD, *n*=6. Data were analyzed using unpaired t-test. PC (phosphatidylcholine), LPC (lysophosphatidylcholine), PE (phosphatidylethanolamine), SM (sphingomyelin), CER (ceramide), PUFA (Polyunsaturated fatty acids).* Plasma ceramide concentration is pmol/µl
